# Syngeneic hematopoietic stem cell transplantation with post-transplantation cyclophosphamide to induce immune tolerance of allogenic skin graft survival in rat

**DOI:** 10.1007/s12026-026-09862-3

**Published:** 2026-10-22

**Authors:** Kaja Kasarełło, Renata Podkowińska-Polak, Piotr Olejnik, Jacek Dziedziak, Robert Wrzesień, Agnieszka Cudnoch-Jędrzejewska, Emilian Snarski

**Affiliations:** 1 Chair and Department of Physiology and Pathophysiology, Laboratory of Centre for Preclinical Research, Medical University of Warsaw, Warsaw, Poland; 2 Department of Pathology, Medical University of Warsaw, Warsaw, Poland; 3 Central Laboratory of Experimental Animals, Medical University of Warsaw, Warsaw, Poland; 4 Institute of Medical Sciences, University of Zielona Góra, Zielona Góra, Poland

**Keywords:** Organ transplantation, Hematopoietic stem cells, Cyclophosphamide, Graft rejection, Immunosuppression

## Abstract

In haploidentical hematopoietic stem cell transplantation (HSCT), the addition of post-transplantation cyclophosphamide (Cy) can help to induce immunotolerance between the donor and haploidentical immune system, preventing graft-versus-host disease between the haploidentical immune system and organs/ tissues of the recipient. We hypothesized that the situation could be reversed and replicated during the transplantation of allogenic tissue and syngeneic (autologous model) HSCT with post-HSCT Cy. We hypothesized that conditioning (pre-HSCT Cy) causes immunoablation of the recipient, and post-HSCT Cy deletes allo-reactive clones from transplanted HSC thus enabling immunotolerance to simultaneously transplanted tissue (organ). To test this hypothesis, we developed a model of autologous HSCT (syngeneic HSCT) with simultaneous transplantation of allogenic tissue in rat. The study rats received: first immunoablation, then allogenic skin graft and syngeneic HSCT, and later post-transplantation Cy. The administration of Cy before and after syngeneic HSCT with simultaneous transplantation of skin graft prolonged skin graft survival from 3 to 12 days and changed the pattern of rejection. The addition of Cy post-HSCT resulted in a statistically significant increase in TNFα secretion before rejection compared to acute rejection without Cy post-HSCT. The sole administration of Cy before and after syngeneic HSCT with simultaneous organ transplantation is not sufficient to achieve full tolerance to allogenic tissue. However, with changes to conditioning or additional immunosuppression, this goal could be achieved.

**Supplementary Information:**

The online version contains supplementary material available at https://doi.org/10.1007/s12026-026-09862-3.

## Introduction

 Allogenic organ transplantation is widely used as a lifesaving treatment in case of organ failure [1]. The critical step after transplantation is the prevention of graft rejection. This requires long-term systemic immunosuppression in the patients after organ transplantation [2]. One way to solve this problem is the transplantation of bone marrow and organ from the same human leukocyte antigen (HLA) identical donor. This type of simultaneous transplantation creates an immune system that tolerates the transplanted organ. In 2006, the report of a series of cases of myeloma patients who received a bone marrow transplantation from a family donor and a kidney from the same donor was published [3]. These recipients did not reject the transplanted organ even after standard immunosuppression for allogenic hematopoietic stem cell transplantation was withdrawn. Moreover, three of the recipients lost chimerism (i.e. the recipient eliminated the donor’s immune system cells), yet they did not develop graft-versus-host disease (GvHD) directed against the transplanted kidney in long-term observation [3, 4]. The effect of persistent immunotolerance after transplantation of an organ and bone marrow from the same donor is attributed to donor microchimerism, the survival of donor immune cells in small quantities that induce immunotolerance of the transplanted organ and do not attack the recipient tissues [5].

Allogeneic hematopoetic stem cell transplantation (HSCT), widely used for the treatment of hematologic disorders, traditionally requires full HLA match, proper conditioning and immunosuppression to achieve immunotolerance and prevent transplant rejection and GvHD [6]. The methodology of allo HSCT was further developed by the addition of post-transplantation cyclophosphamide (Cy) that can prevent GvHD even in a haploidentical setting [7], thus enabling transplantation of only partially matched donors. The action of Cy here is based on the induction of apoptosis in T lymphocytes, which demonstrate activity against host antigens after bone marrow transplantation. Interestingly, when after haploidentical HSCT the immunosuppression is withdrawn the donor immune system tolerates all recipient organs and tissues although they are only half identical in HLA antigens [8]. This proves that lifelong immunosuppression is not needed for long-term prevention of graft rejection when the immune system is only haploidentical to organs.

The elimination of autoreactive lymphocyte clones by chemotherapy is the basis for the use of autologous HSCT in autoimmune diseases that do not respond to standard immunosuppressive treatment [9]. After autologous HSCT the new immune system has to form just like after allogenic HSCT [10]. We hypothesized that if transplantation of allogenic tissue is performed at the time of development of a new immune system after autologous HCST, the addition of post-transplantation Cy would induce apoptosis of autologous lymphocyte clones reactive to allogenic tissue which would later lead to graft tolerance without the need for additional immunosuppression. This would mimic the situation observed in haploidentical HSCT, the destruction of the lymphocyte clones reactive to haploidentical graft tissue during the development of a new autologous immune system.

In our previous work, we performed syngeneic HSCT in rats with experimental allergic encephalomyelitis, an animal model of multiple sclerosis, as a model of autologous HSCT. Littermates were used as the hematopoetic stem cells (HSC) donors, to ensure the closest genetic identity, and creating the model of autologous HSCT by use of syngeneic cells. Those experiments provided a rationale to use the post-HSCT Cy, as it decreased the disease progression intensity after syngeneic HSCT in rats [11]. We decided to implement this model of post-transplantation Cy in the presented study, to eliminate syngeneic alloreactive lymphocytes and achieve immunotolerance of allogenic skin graft. This hypothesis and animal model were developed in 2015 independently of a “first-in-human” study of Chruscinski et al. in which the autologous HSCT was used with success to treat recurrent autoimmune disease of the liver and induce tolerance to liver graft, thus providing later proof that this concept works in human if myeloablative conditioning is used [12].

In this exploratory study, we show results of syngeneic HSCT (model of autologous HSCT) after immunoablative conditioning, performed together with allogeneic skin transplantation in the rat with post-HSCT Cy to induce immunotolerance of the transplanted organ.

## Materials and methods

### Animals

Experiments were conducted using rats from the Animal House of the Medical University of Warsaw. 12-week-old male Sprague Dawley (SPRD) inbreed rats were used in the study as the recipients of the transplant. Male rats from the same litter were used as the donors of syngeneic HSC. 12-week-old male spontaneously hypertensive rats (SHR) inbreed rats were used as the donors of the allogenic skin grafts. Experiments were performed according to II Warsaw Local Ethics Comittee acceptance (Act no. 57/2015, with corrections 066/2016, and WAW2/188/2019). Animals were housed in pairs in standard cages up to the transplantation, and individually after transplantation. Stable temperature (20–24 °C) and light (light: dark, 12:12) conditions were provided. Water and food were supplied *ad libitum*.

### Experiment design

Experimental SPRD rats received intraperitoneally pre-transplantation immunoablative conditioning (Cy, 125 mg/kg, Baxter) on day − 2. On day 0 rats received simultaneous transplantation of syngeneic HSCT (1 × 10^7^ cells in 0.5 ml PBS, intravenously) and allogenic skin graft. On day + 3 and + 4 rats received additional intraperitoneal chemotherapy (Cy, 20 mg/kg, Baxter). Doses were chosen based on our previous experiments [11]. Control animals received only skin transplantation, or pre-transplantation conditioning (Cy, 125 mg/kg) followed by skin transplantation with syngeneic HSCT without post-transplantation conditioning. At different time points (specified below) rats were euthanized by subjection to general anaesthesia by intraperitoneal injection of 100 mg/kg ketamine and 10 mg/kg xylazine (Vetoquinol, Biowet) and subsequent spinal cord severance.

The pilot study was performed to assess the time of skin graft survival. Rats were subjected to the procedures described above and were observed until the graft rejection. After the transplantation, the condition of the skin grafts was assessed daily by an independent experimenter in a blinded fashion. After visible tissue necrosis (hardening of the grafted tissue, changing color into dark/black), grafts were considered completely rejected [13]. After all the grafts were rejected rats were euthanized.

**Fig. 1:**
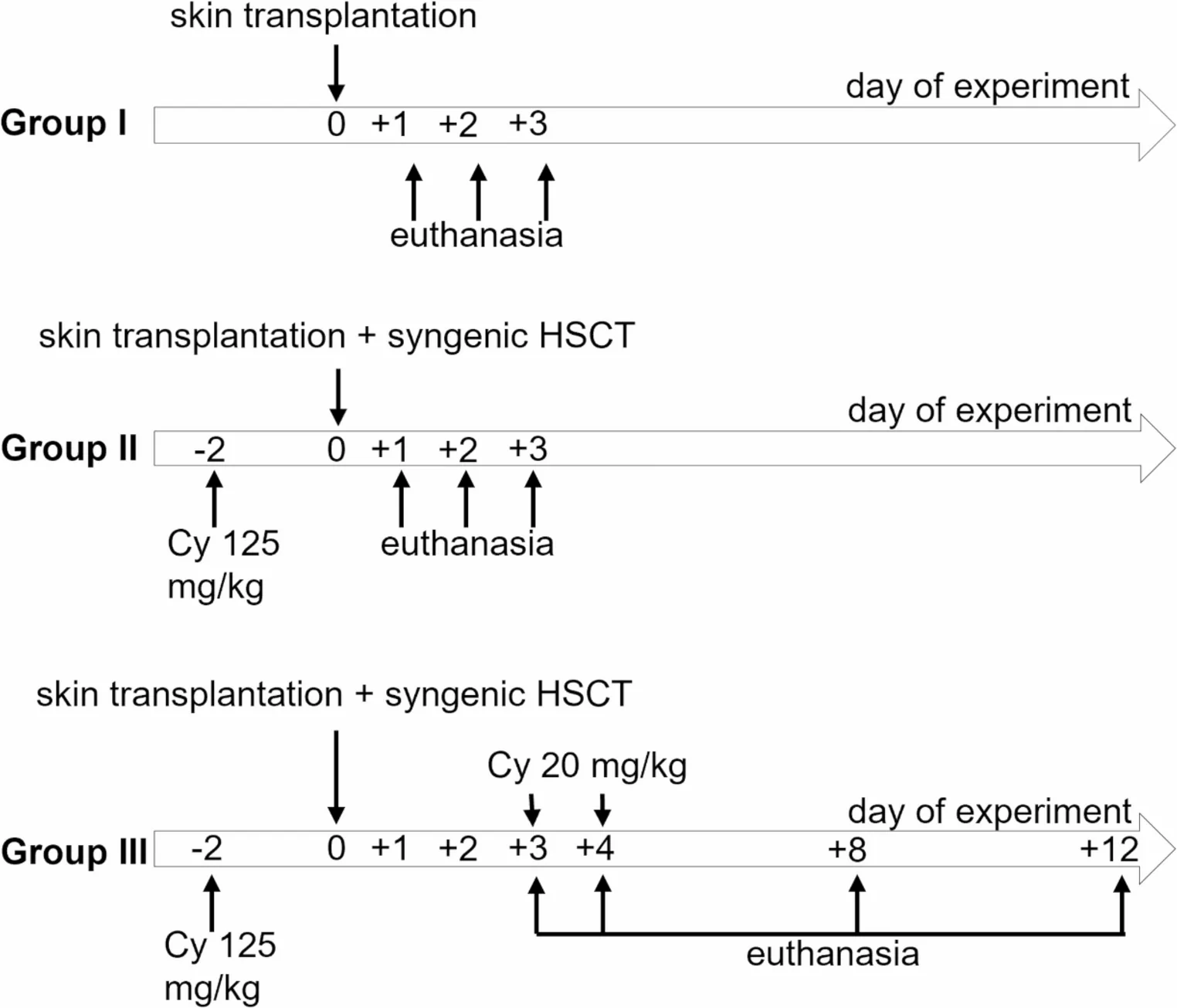
Schedule of the main experiment. The pilot experiments have shown full graft rejection on day + 3 in group I and II, and graft rejection and necrosis on day + 13 in group III. The groups II and III had identical treatment until day + 3 when group III received post-transplantation cyclophosphamide

The main experiment was scheduled based on the results from the pilot study. Hence, the animals from the control groups were euthanized on the days + 1, +2, and + 3 after skin transplantation, and animals from the experimental group were euthanized on the days + 3, +4, + 8, and + 12 after skin transplantation and syngeneic HSCT. Directly after euthanasia the skin grafts were collected and stored upon analysis. The time schedule of the main experiment is presented in Fig. 1.

### Skin graft collection and transplantation

Allogenic skin grafts were obtained from SHR rats. Donors were subjected to general anaesthesia by intraperitoneal injection of 100 mg/kg ketamine and 1 mg/kg xylazine (Vetoquinol, Biowet), and their back was disinfected and shaved. Next, 0.5 × 0.5 cm skin fragments from the back of the animals were cut off using a surgical blade. One rat served as the donor of about 10 grafts. Anesthetized donor rats were euthanized after graft collection by spinal cord severance. Graft recipients were subjected to general anaesthesia by intraperitoneal injection of 100 mg/kg ketamine and 10 mg/kg xylazine (Vetoquinol, Biowet), and following disinfection and shaving, 0.5 × 0.5 cm skin fragment at the top of their neck was cut off. Next, the allogenic skin fragment from the donor was sutured using a soluble surgical suture. After the surgery, the recipient animals received intraperitoneally an analgesic (metamizole sodium, 15 mg/kg, Biovetalgin, Biowet) and subcutaneously the antibiotic (enrofloxacin, 10 mg/kg).

### Hematopoietic stem cell collection and transplantation

Syngeneic hematopoetic stem cells were obtained from the SPRD rats from the same litter as the transplant recipients, for the possible closest genetic identity, to mimic the autology of the transplant. Donors were subjected to general anaesthesia by intraperitoneal injection of 100 mg/kg ketamine and 10 mg/kg xylazine (Vetoquinol, Biowet), and euthanized by spinal cord severance. Long bones were collected, and after removal of the capitulum, medullary canals were flushed with as small as possible volume of PBS (about 0.25 ml per bone). The obtained marrow was next filtered using a 70 µl cell strainer (Falcon, Corning). Cells were diluted to obtain a suspension of 1 × 10^7^/ 0.5 ml PBS.

Recipients remaining under general anaesthesia after skin transplantation were next placed on the hot plate to dilate the tail veins. After a few minutes, the cell suspension of 1 × 10^7^/ 0.5 ml PBS was injected into the tail vein of the rats.

### Analysis

#### Tissue collection

After the animal euthanasia, the skin grafts with the 0.3 cm margin of recipient skin were collected. Next, the tissue was either immediately frozen for immunoenzymatic analysis or fixed in 4% paraformaldehyde (PFA) for histopathological analysis.

#### Histopathological analysis of skin grafts

Fixed tissues were routinely processed. Tissues embedded in paraffin were cut into 3 μm slices using a microtome (Leica, Germany). Slides were stained according to standard protocol with haematoxylin and eosin and examined microscopically. Images were captured using the Aperio GT 450 DX scanner (Leica BioSystems).

#### Immunoenzymatic analysis of TNFα, CD3 and Caspase 3 levels in skin graft homogenates

Collected skin grafts were stored upon analysis at -80 °C. Before the analysis tissues were thawed, weighted, and homogenized in a calculated volume of ice-cold PBS to obtain 20% homogenates, at 50 Hz for 20 min (TissueLyser, Qiagen, Germany). Homogenates were then subjected twice to the thaw-freeze cycle to rupture the cell membrane, and centrifuged (10,000 rpm, 10 min, 4 °C). TNFα, CD3 and caspase 3 levels were analyzed in obtained homogenates. Commercially available ELISA kits were used, including Rat TNFα ELISA kit (Wuhan Fine Biotech Co.), Rat CD3 ELISA kit (Wuhan Fine Biotech Co.) and Rat Casp3 ELISA kit (Wuhan Fine Biotech Co.). Analysis was performed according to the manufacturer’s manuals. Microplates were read at a wavelength of 450 nm using a microplate reader (Microplate Reader, Bio-Rad, USA). Optical density (OD values) was determined and presented in figures.

#### Statistical analysis

Continuous variable distribution was assessed using the Shapiro–Wilk test. Variables were reported as mean and the standard deviation (SD), when normally distributed, otherwise the median and the 25th and 75th percentile (Q1 and Q3) were calculated. The ANOVA test with Sidak’s post-hoc test was used for the comparison of the normally distributed continuous variables, otherwise, the Kruskal–Wallis test with Dunn’s post-hoc test was used. The significance level was set at 0.05. The statistical significance was marked on the figures as follows: *p < 0.05; **p < 0.01; ***p < 0.001; ****p < 0.0001. Analysis was performed using Graph-Pad Prism software.

## Results

### Duration of skin graft survival

The pilot study was conducted to assess the time of skin graft survival. We observed, that in control groups, both without any conditioning and with pre-transplantation immunoablative conditioning (Cy), skin grafts were rejected on the 3rd day after simultaneous transplantation with syngeneic HSCT. The administration of an additional post-transplantation dose of Cy prolonged the graft’s survival for 9 days, up to day 12 (Table 1). Results obtained in the pilot study were used for the design of the main study. Based on those we planned the time points during graft survival upon its rejection, for skin graft collection for the histopathological and immunoenzymatic analysis.

**Table 1:** Duration of skin graft survival in rats subjected to skin transplantation with HSCT. Number of animals, *n* = 3

	pre-transplantation conditioning (Cy, 125 mg/kg) (immunoablation)	skin transplantation +HSCT	post-transplantation conditioning (Cy, 20 mg/kg) (immunomodulatory removal of alloreactive clones)	graft rejection
Group I, *n* = 3		day 0		day + 3
Group II, *n* = 3	day − 2	day 0		day + 3
Group III, *n* = 3	day − 2	day 0	day + 3, day + 4	day + 12

### Histopathological analysis

Indicators of graft rejection were assessed on tissue sections. The presence of the following was analyzed: apoptotic cells/ dyskeratotic cells (AC/ DC), chronic inflammation (CI), stromal inflammation (SI), basal vacuolar change (BVC), spongiosis, microvesicles, clefts (SMC), separation of epidermis (SoE), necrosis (N) and acute inflammation (AI).

In general the histopathological analysis based on morphological criteria revealed a very similar picture in all the skin grafts examined (Table 2). The histopathological features are nonspecific, and can be caused by normal mechanisms of damaged tissue repair. Every indicator of graft rejection was found in the tissue sections at least once. Apoptotic/ dyskeratotic cell was noted only on one slide from a rat from group III euthanized on day + 12. The basal vacuolar change was only reported on one slide from a rat from group I euthanized on day + 2. Separation of epidermis was noted on slides from rats from group II, one euthanized on day + 1, and three from rats euthanized on day + 2. In opposite, no spongiosis, microvesicles, or clefts were observed on slides from rats from group II. Those were visible on slides from rats from group I euthanized on days + 2 and + 3, and rats from group III euthanized on days + 8 and + 12. Other indicators (chronic inflammation, stromal inflammation, necrosis and acute inflammation) were present in almost all the slides with very few exceptions. Noticeable differences were observed for chronic inflammation, where greatest intensity was present for slides from rats from group I, and alike decrease in intensity was observed on slides from rats from groups II and III. On the other hand, necrosis and acute inflammation were most intense on slides from rats from group III. Individual values of graft rejection indicators were available in supplementary materials (Supp. 1).

**Table 2:** Graft rejection indicators. Calculated means for each experimental group. 0 - absence, 1 - presence. For CI: 0 – absence, 1 – small (1–10% vessels with inflammatory infiltrations), 2 – medium (11–50% vessels with inflammatory infiltrations), 3 – high (> 50% vessels with inflammatory infiltrations). *Apoptotic cells/ dyskeratotic cells (AC/ DC)*,* chronic inflammation (CI)*,* stromal inflammation (SI)*,* basal vacuolar change (BVC)*,* spongiosis*,* microvesicles*,* clefts (SMC)*,* separation of epidermis (SoE)*,* necrosis (N) and acute inflammation (AI).* Number of grafts: *n* = 6, except group I + 3 where *n* = 5 and group III + 12 where *n* = 8

		AC/DC	CI	SI	BVC	SMC	SoE	N	AC
Group I	day + 1	0	1	1	0	0	0	1	1
	day + 2	0	1,17	1	0,08	0,42	0	0,83	0,83
	day + 3	0	1,25	0	0	0,60	0	0,80	0,80
Group II	day + 1	0	0,50	0,42	0	0	0,17	1	1
	day + 2	0	0,42	0,83	0	0	0,50	0,67	0,67
	day + 3	0	0,67	1	0	0	0	1	1
Group III	day + 3	0	0,42	1	0	0	0	1	1
	day + 4	0	0,67	0,83	0	0	0	1	1
	day + 8	0	0,58	1	0	0,17	0	1	1
	day + 12	0,13	0,75	1	0	0,25	0	1	1

**Fig. 2:**
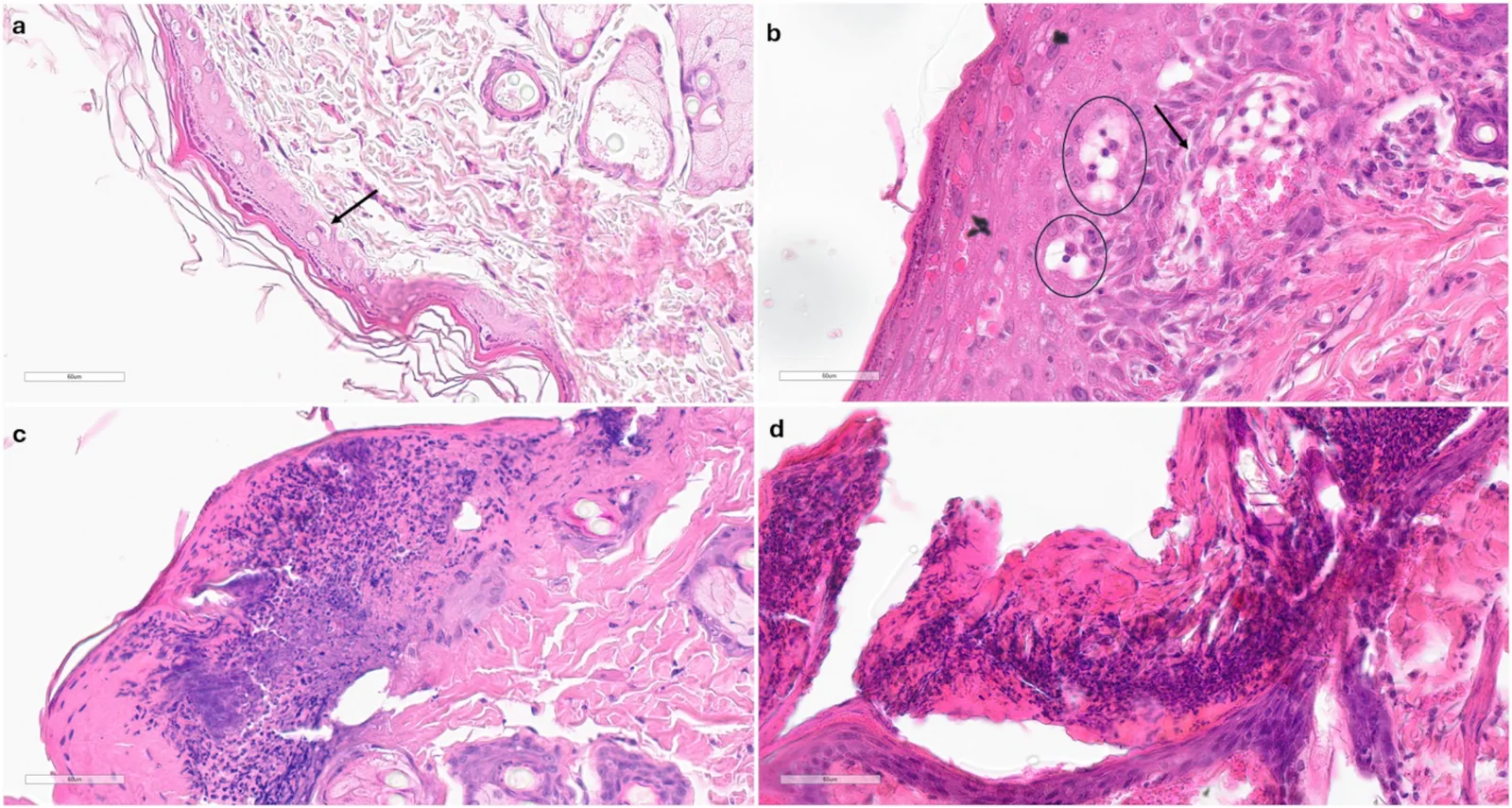
Representative morphological changes in skin sections from rats subjected to skin transplantation and HSCT. H + E staining. Apoptotic cell indicated by arrow (**a**), spongiosis indicated by arrow, microvessels in circles (**b**), ulceration (**c**), fibrinous and purulent exudate (**d**)

In Fig. 2 an illustration of the representative indicators of skin graft rejection that were observed on slides is presented. In Fig. 2a an apoptotic cell is indicated with the visible shrunken hyperchromatic nucleus and eosinophilic cytoplasm. The spongiosis with microvesicle formation is presented in Fig. 2b Ulceration and epidermal necrosis with fibrinopurulent exudate are visible throughout Fig. 2c Fibrinous and purulent exudate is also presented in Fig. 2d.

### Immunoenzymatic analysis

**Fig. 3:**
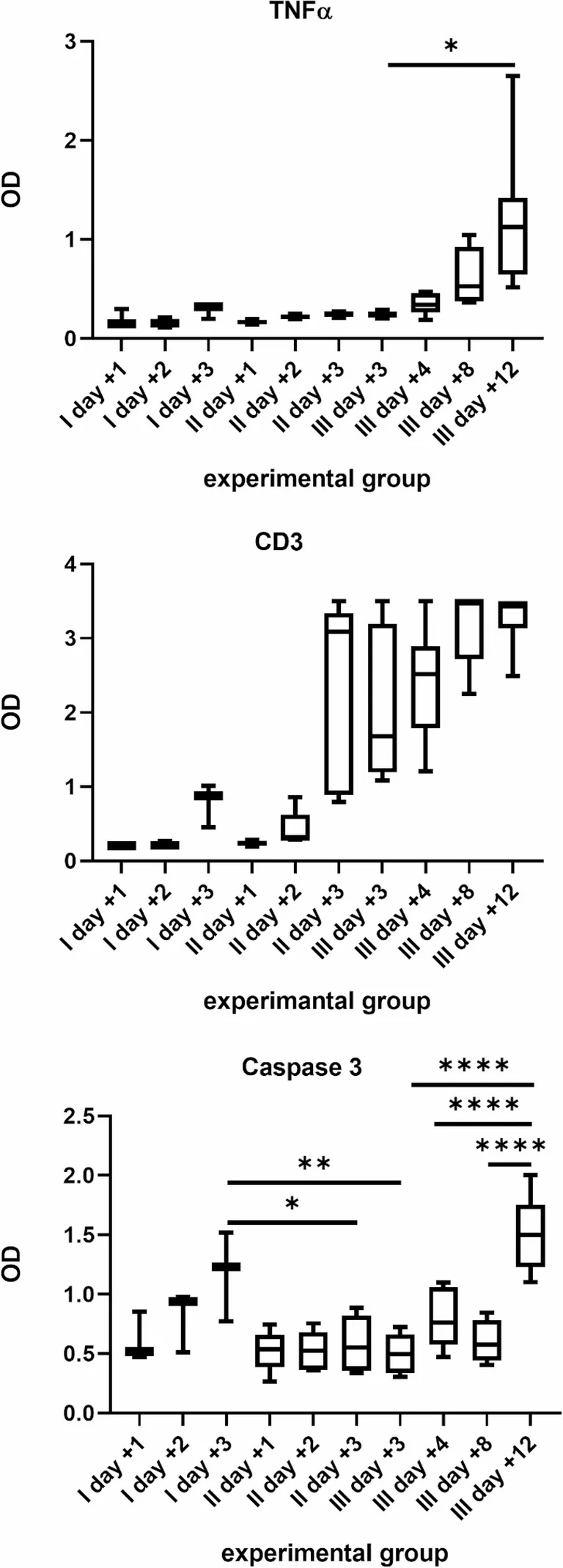
TNFα, CD3 and caspase 3 levels in skin graft homogenates from rats. Box plot presenting the median (horizontal line), the box covers values from the first to the third quartile and whiskers from the quartiles to the minimum and maximum values. *OD - optical density*. The ANOVA test was performed for Caspase 3 level statistical analysis, the Kruskal-Wallis test was performed for TNFα and CD3 levels statistical analysis. *p < 0.05; **p < 0.01; ***p < 0.001; ****p < 0.0001. Number of biological replicates: TNFα: *n* = 3 [I day + 1, I day + 2, I day + 3]; *n* = 5 [III day + 8]; *n* = 6 [II day + 1, II day + 2, II day + 3, III day + 3, III day + 4]; *n* = 12 [III day + 12]. CD3: *n* = 3 [I day + 1, I day + 2, I day + 3]; *n* = 5 [II day + 2, II day + 3, III day + 3, III day + 8]; *n* = 6 [II day + 1, III day + 4]; *n* = 11 [III day + 12]. Caspase 3: *n* = 3 [I day + 1, I day + 2, I day + 3]; *n* = 4 [III day + 8]; *n* = 5 [II day + 3, III day + 3]; *n* = 6 [II day + 1, II day + 2, III day + 4]; *n* = 12 [III day + 12]

Levels of TNFα, as the main proinflammatory cytokine indicating the intensity of inflammatory response, CD3 as the marker of antigen-specific immune cells (lymphocytes) presence and caspase 3 as the marker of apoptosis were analyzed in skin graft homogenates (Fig. 3).

TNFα levels were similar in skin graft homogenates from rats from groups I and II, and from the rats from group III euthanized on days + 3 and + 4. The TNFα level began to increase in rats from group III euthanized on day + 8 and was significantly higher on day + 12 compared to day + 3 (p-value: 0,0219). .

Levels of CD3 were similar in homogenates of skin grafts from rats from group I, and in rats euthanized on days + 1 and + 2 from group II. Elevated levels of CD3 in skin graft homogenates from rats from group II euthanized on day + 3 and in skin graft homogenates from all rats from group III was observed .

Caspase 3 levels remained at similar levels in all skin graft homogenates, except for the homogenates from rats from group I euthanized on day + 3, where significantly increased levels od Caspase 3 were observed compared to rats from groups II and III euthanized on day + 3 (p-value: 0,0257; p-value: 0,0063 respectively). Additionally, the increase of the caspase 3 level was observed in homogenates from group III rats euthanized on day + 12, compared to those form group III euthanized on day + 3 (p-value: <0,0001), day + 4 (p-value: <0,0001), and day + 8 (p-value: <0,0001).

## Discussion

The immunoablation with cyclophosphamide and syngeneic HSCT failed to induce any change in the pattern of rejection of allogenic skin. The addition of immunomodulatory post-HSCT Cy on days + 3 and + 4 altered the pattern of rejection of the skin graft. However, grafts were rejected by the recipients eventually. Graft rejection and inflammation markers analyzed both in histopathological slices and with immunoenzymatic methods were present in all compared groups and confirmed graft rejection and presence of inflammation, which was increasing during the graft survival time until the graft was finally rejected, although the patterns of rejection differed between the groups.

The CD3/TNFα/Caspase 3 measurements show that HSCT with Cy changes the rejection pattern of the skin graft. Without syngeneic HSCT, graft rejection takes place within 3 days after transplantation, with an increase in CD3 cell and caspase 3 presence in the graft. Syngeneic HSCT with Cy given as conditioning changes this pattern, and a significant increase of CD3 lymphocytes is present in transplanted tissue but no caspase 3 increase is noted. When Cy is added post syngeneic HSCT this pattern changes. The skin graft is infiltrated with CD3 + lymphocytes, but only after another week, the rejection takes place with a significant increase in TNFα levels in tissue. That early rejection could be explained by the toxicity of cells infiltrating tissue after syngeneic HSCT. We speculate, that the addition of post-transplantation Cy changes that pattern, as the infiltrating cells may not destroy tissue directly, but by activating other cells. Such activation may take a longer time, resulting in increased TNFα levels just before the graft rejection in group III. This hypothesis requires further investigation including the assessment of subtypes of infiltrating cells, their activation status or their allo-specificity.

The results of this work stay in contrast with the first human clinical study by Chruscinski et al. where authors achieved immunotolerance of the transplanted liver by autologous HSCT [12]. What differs those two studies is conditioning. We used non-myeloablative doses of Cy whereas Chruscinski used a much stronger myeloablative protocol of busulfan, Cy and anti-thymocyte globulin (ATG). Those approaches reflect the results of early studies in transplantation in human autoimmune diseases. The sole conditioning with Cy failed to achieve sufficient responses [14]. The addition of ATG (or alemtuzumab) provided a deeper reset of the immune system which was needed for achieving deep and long-lasting responses [15]. This shows the potential development of protocol in animal model, either by the addition of busulfan, ATG and/ or alemtuzumab, that could potentially induce long-term tolerance to transplanted organs as observed in the first trial in liver recipients This could also explain how the myeloablative conditioning allowed the tolerance of allogenic marrow and kidney graft recipients without the full donor chimerism [3].

There are several limitations of our study. First, we used skin graft. The skin graft is most likely to be rejected because of the extremely high immunity of the skin tissue. It is possible that less immunogenic tissues such as the liver or kidney would not be rejected in this model. However, if the proposed protocol worked in this model it would very likely work in other types of tissue/organ transplantation. Other limitation was the use of a syngeneic model of HSCT. The syngeneic model of HSCT in rats is easier to perform and can be almost synonymous with autologous in inbred animals, especially when the littermates are used as the donors. However, we cannot exclude that minor genetic variations could possibly impact the results of the study. Another important factor was a relatively small number of animals used. We wanted to limit the number of animals used in experiments to the lowest possible due to the ethical concerns. We achieved this by using some data from pilot experiments we performed as controls in this model (e.g. conditioning dose of cyclophosphamide). Due to the consistency of the presented analysis showing a lack of efficiency of the proposed treatment we performed only histopathological evaluation and analysis of a few markers using the immunoenzymatic method. We withdrew further analysis, i.e. immunohistochemical, while there was no basis for those. Otherwise, further analysis of the subpopulation of infiltrating cells would be beneficial. After the clinical data from other group were presented we decided not to include more animals as it was clear that changes in the protocol would be needed to achieve possible immune tolerance of skin graft. Those could include either changes to conditioning, timing of post-transplantation Cy, addition of ATG and/or long-term calcineurin inhibitors. Summarizing, it is likely that spontaneous immunotolerance that is seen in some of the organ recipients could be replicated by autologous HSCT with conditioning stronger than sole Cy, as insufficient myeloablation may contribute to the failure of graft tolerance induction [12]. For further explanation of underlying mechanisms analysis of immune ablation/reconstitution with engraftment studies would help to explain whether the effects are only based on Cy activity, or immune tolerance was induced.

Our results provide a model for studying syngeneic, mimicking autologous HSCT with organ transplantation in rats. Moreover, based on the results obtained, we hypothesize, that syngeneic HSCT preceded by Cy, and with additional post-transplantation Cy is unable to eliminate cells responsible for rejection of the graft and that the addition of other drugs (e.g. ATG or alemtuzumab) or immunosuppression is needed in further experiments.

To conclude, the cyclophosphamide conditioning followed with syngeneic HSCT and post-transplantation cyclophosphamide failed to induce tolerance of simultaneous allogeneic tissue graft. However, the procedure altered the rejection pattern and opened a possibility that the addition of ATG or other immunosuppressants could provide immunotolerance, as it was shown in humans.

## Supplementary Information

Below is the link to the electronic supplementary material.

Supplementary Material 1 (DOCX 22.2 KB)

## Data Availability

The data that support the findings of this study are available from the corresponding author (KK) upon request and partially within the article supplementary materials.
