# Supplementary material for: Syngeneic hematopoietic stem cell transplantation with post-transplantation cyclophosphamide to induce immune tolerance of allogenic skin graft survival in rat
Source: Immunol Res. 2026 Oct 22;74(1):129. doi: 10.1007/s12026-026-09862-3 (PMC13690888; doi:10.1007/s12026-026-09862-3)
Supplement: Supplementary file 1 [file 12026_2026_9862_MOESM1_ESM.docx]

Supplementary materials

Supp. 1. Graft rejection indicators. Values obtained from each slide (from each rat). *Apoptotic cells/ dyskeratotic cells (AC/ DC), chronic inflammation (CI), stromal inflammation (SI), basal vacuolar change (BVC), spongiosis, microvesicles, clefts (SMC), separation of epidermis (SoE), necrosis (N) and acute inflammation (AI). 0 - absence, 1 - presence. For CI: 0 – absence, 1 – small (1-10% vessels with inflammatory infiltrations), 2 – medium (11-50% vessels with inflammatory infiltrations), 3 – high (>50% vessels with inflammatory infiltrations).*

|  |  | AC/ DC | CI | SI | BVC | SMC | SoE | N/U | AC |
| --- | --- | --- | --- | --- | --- | --- | --- | --- | --- |
| Group I | day +1 | 0 | 1 | 1 | 0 | 0 | 0 | 1 | 1 |
|  |  | 0 | 1 | 1 | 0 | 0 | 0 | 1 | 1 |
|  |  | 0 | 1 | 1 | 0 | 0 | 0 | 1 | 1 |
|  |  | 0 | 1 | 1 | 0 | 0 | 0 | 1 | 1 |
|  |  | 0 | 1 | 1 | 0 | 0 | 0 | 1 | 1 |
|  |  | 0 | 1 | 1 | 0 | 0 | 0 | 1 | 1 |
|  | day +2 | 0 | 1 | 1 | 0 | 0 | 0 | 1 | 1 |
|  |  | 0 | 1 | 1 | 0 | 1 | 0 | 0 | 0 |
|  |  | 0 | 1 | 1 | 0 | 1 | 0 | 1 | 1 |
|  |  | 0 | 1 | 1 | 0 | 0 | 0 | 1 | 1 |
|  |  | 0 | 1 | 1 | 0,5 | 0 | 0 | 1 | 1 |
|  |  | 0 | 2 | 1 | 0 | 0,5 | 0 | 1 | 1 |
|  | day +3 | 0 | 2 | 1 | 0 | 1 | 0 | 1 | 1 |
|  |  | 0 | 1 | 1 | 0 | 0 | 0 | 1 | 1 |
|  |  | 0 | 1 | 1 | 0 | 1 | 0 | 1 | 1 |
|  |  | 0 | 1 | 1 | 0 | 0 | 0 | 1 | 1 |
|  |  | 0 | 1/2. | 1 | 0 | 1 | 0 | 0 | 0 |
| Group II | day +1 | 0 | 0,5 | 0 | 0 | 0 | 0 | 1 | 1 |
|  |  | 0 | 0,5 | 0 | 0 | 0 | 0 | 1 | 1 |
|  |  | 0 | 0,5 | 1 | 0 | 0 | 0 | 1 | 1 |
|  |  | 0 | 0,5 | 0,5 | 0 | 0 | 0 | 1 | 1 |
|  |  | 0 | 0,5 | 0 | 0 | 0 | 0 | 1 | 1 |
|  |  | 0 | 0,5 | 1 | 0 | 0 | 1 | 1 | 1 |
|  | day +2 | 0 | 0 | 1 | 0 | 0 | 1 | 1 | 1 |
|  |  | 0 | 1 | 1 | 0 | 0 | 0 | 0 | 0 |
|  |  | 0 | 0,5 | 0 | 0 | 0 | 0 | 0 | 0 |
|  |  | 0 | 0 | 1 | 0 | 0 | 1 | 1 | 1 |
|  |  | 0 | 0,5 | 1 | 0 | 0 | 0 | 1 | 1 |
|  |  | 0 | 0,5 | 1 | 0 | 0 | 1 | 1 | 1 |
|  | day +3 | 0 | 1 | 1 | 0 | 0 | 0 | 1 | 1 |
|  |  | 0 | 1 | 1 | 0 | 0 | 0 | 1 | 1 |
|  |  | 0 | 0,5 | 1 | 0 | 0 | 0 | 1 | 1 |
|  |  | 0 | 0,5 | 1 | 0 | 0 | 0 | 1 | 1 |
|  |  | 0 | 0,5 | 1 | 0 | 0 | 0 | 1 | 1 |
|  |  | 0 | 0,5 | 1 | 0 | 0 | 0 | 1 | 1 |
| Group III | day +3 | 0 | 0,5 | 1 | 0 | 0 | 0 | 1 | 1 |
|  |  | 0 | 1 | 1 | 0 | 0 | 0 | 1 | 1 |
|  |  | 0 | 0 | 1 | 0 | 0 | 0 | 1 | 1 |
|  |  | 0 | 0,5 | 1 | 0 | 0 | 0 | 1 | 1 |
|  |  | 0 | 0,5 | 1 | 0 | 0 | 0 | 1 | 1 |
|  | day +4 | 0 | 0,5 | 1 | 0 | 0 | 0 | 1 | 1 |
|  |  | 0 | 1 | 1 | 0 | 0 | 0 | 1 | 1 |
|  |  | 0 | 0,5 | 1 | 0 | 0 | 0 | 1 | 1 |
|  |  | 0 | 1 | 1 | 0 | 0 | 0 | 1 | 1 |
|  |  | 0 | 0 | 0 | 0 | 0 | 0 | 1 | 1 |
|  |  | 0 | 1 | 1 | 0 | 0 | 0 | 1 | 1 |
|  | day +8 | 0 | 0,5 | 1 | 0 | 1 | 0 | 1 | 1 |
|  |  | 0 | 1 | 1 | 0 | 0 | 0 | 1 | 1 |
|  |  | 0 | 0,5 | 1 | 0 | 0 | 0 | 1 | 1 |
|  |  | 0 | 0,5 | 1 | 0 | 0 | 0 | 1 | 1 |
|  |  | 0 | 0,5 | 1 | 0 | 0 | 0 | 1 | 1 |
|  |  | 0 | 0,5 | 1 | 0 | 0 | 0 | 1 | 1 |
|  | day +12 | 1 | 0,5 | 1 | 0 | 1 | 0 | 1 | 1 |
|  |  | 0 | 1 | 1 | 0 | 0 | 0 | 1 | 1 |
|  |  | 0 | 1 | 1 | 0 | 0 | 0 | 1 | 1 |
|  |  | 0 | 1 | 1 | 0 | 0 | 0 | 1 | 1 |
|  |  | 0 | 0,5 | 1 | 0 | 0 | 0 | 1 | 1 |
|  |  | 0 | 1 | 1 | 0 | 0 | 0 | 1 | 1 |
|  |  | 0 | 0,5 | 1 | 0 | 0 | 0 | 1 | 1 |
|  |  | 0 | 0,5 | 1 | 0 | 1 | 0 | 1 | 1 |
